# From small brains to smart machines: translating *Caenorhabditis elegans* neural circuits into artificial intelligence

**DOI:** 10.3389/fncir.2026.1731513

**Published:** 2026-03-09

**Authors:** He Liu, Panpan Zheng, Xuebin Wang

**Affiliations:** 1 Department of Systems Science, Faculty of Arts and Sciences, Beijing Normal University, Guangdong, Zhuhai, China; 2 School of Systems Science, Beijing Normal University, Beijing, China; 3 Guangdong Institute of Intelligence Science and Technology, Guangdong, Zhuhai, China

**Keywords:** artificial intelligence, artificial neural network, bionics design, *Caenorhabditis elegans*, neural circuits

## Abstract

The hermaphroditic *Caenorhabditis elegans*, with its fully mapped connectome of 302 neurons, offers a paradigmatic example of how a minimal nervous system governs biotic, adaptive, and context-dependent behaviors. In contrast, modern artificial intelligence systems achieve intelligence through scale rather than efficiency, relying instead on massive datasets and artificially engineered architectures. This mini-review explores how *Caenorhabditis elegans* neural circuits can inform the development of more efficient and flexible artificial neural networks. We highlight recent studies that translate the principles inherent to *Caenorhabditis elegans* neural circuits into artificial neural network architectures, with applications in machine control and image classification, resulting in enhanced robustness and improved performance. By distilling neural principles from the simplest known nervous system, this mini-review outlines a pathway toward compact, adaptive, and biologically inspired artificial intelligence systems.

## 1 Introduction

Despite the remarkable achievements of deep learning in perception, language, and decision-making, current artificial intelligence (AI) systems remain fundamentally different from biological systems in their mechanisms (Luo, 2021; He et al., 2016; Yoshimura et al., 2020). Deep architectures of artificial neural networks (ANNs) typically require vast amounts of labeled data, enormous computational resources, and extensive parameter tuning to achieve high performance. Once trained, they often lack flexibility, struggling to generalize beyond the training distribution or adapt to novel environments without retraining (LeCun et al., 2015; Goodfellow et al., 2016; Bengio et al., 2009). Moreover, most ANNs are static in structure and rely on backpropagation that has no known biological counterpart. In contrast, even tiny nervous system, such as that of hermaphroditic *Caenorhabditis elegans* with only 302 neurons, achieve robust, context-dependent behaviors, lifelong adaptability, and efficient learning from sparse experience (Yuste, 2015; Farisenkov et al., 2022; Gibbons et al., 2022). The biological systems operate under severe energy and wiring constraints, yet they maintain stability, plasticity, and resilience through local learning rules, neuromodulation, and recurrent dynamics. Understanding the principles of such biological nervous system provides not only mechanistic insight into natural intelligence but also a promising foundation for designing new AI system architectures that are efficient, adaptive, and interpretable (Lechner Mathias et al., 2020; Wang et al., 2025a; Bardozzo et al., 2024, 2025; Fiore et al., 2025; Zhao et al., 2024). Bridging these two domains may help overcome some of the key limitations of current deep learning and move toward more brain-like artificial systems.

The hermaphroditic *Caenorhabditis elegans* provides a uniquely powerful model to bridge the structure of biological nervous system with the design of AI system. Its entire nervous system, consisting of precisely 302 neurons and approximately 7,000 synaptic connections, has been mapped at single-cell and synaptic resolution, representing one of the few fully resolved connectomes currently available for any animal species (Cook Steven et al., 2019; Witvliet et al., 2021; Varshney et al., 2011; Lin et al., 2024). Despite this simplicity, *Caenorhabditis elegans* exhibits a rich behavioral repertoire, including navigation, foraging, learning, and decision-making, all orchestrated by compact yet dynamic neural circuits (Bargmann, 2006; Emmons, 2018; Gabel et al., 2007; Faumont et al., 2012; Rahmani and Chew, 2021). Each neuron has a well-characterized morphology, connectivity, and neurotransmitter profile, allowing precise modeling of circuit function and plasticity. Moreover, the neural circuit architectures display properties reminiscent of efficient AI systems: sparse and modular organization, recurrent loops and small-world connectivity that balance efficiency with performance (Wang et al., 2025b,c). Because its neural activity and behavior can be measured and manipulated *in vivo* with high precision, *Caenorhabditis elegans* provides an ideal minimal model to uncover generalizable design principles of biological neural structure. These principles, local learning, distributed control, and adaptive modulation, can in turn inspire the development of novel AI system architectures that integrate structural efficiency with functional flexibility.

The goal of translating *Caenorhabditis elegans* nervous system into AI system is not to replicate its anatomy neuron by neuron, but to extract the functional principles that underlie its computation. The worm's nervous system demonstrates how complex and adaptive behaviors can emerge from minimal circuitry through distributed processing, feedback control, and context-dependent modulation (Thomas et al., 1993; Metaxakis et al., 2018; Ghosh et al., 2017). Rather than copying the exact connectome, ANNs research can draw inspiration from how *Caenorhabditis elegans* neural network organize information flow, integrate sensory input, and regulate learning via neuromodulators. These principles suggest alternative approaches to ANN design, such as modular and recurrent architectures, local learning rules, and dynamic gain control, that emphasize efficiency and adaptability over scale. By understanding how a small biological nervous system achieves generalization, robustness, and flexibility, we can begin to design AI systems that learn and reason more like living organisms: grounded in interaction, feedback, and plasticity rather than brute-force computation.

To translate the neural principles of the *Caenorhabditis elegans* nervous system into ANN design, a fundamental prerequisite is to elucidate the organization and operation of its neural circuits. The *Caenorhabditis elegans* connectome offers a comprehensive and dynamic blueprint of information flow, unveiling how simple structural motifs regulate complex, adaptive behaviors (Ripoll-Sánchez et al., 2023; Flavell and Gordus, 2022). The subsequent section highlights key architectural and computational features of this compact nervous system, with the potential to inform the development of more efficient and flexible ANNs.

## 2 Overview of *Caenorhabditis elegans* nervous system

**Figure 1:**
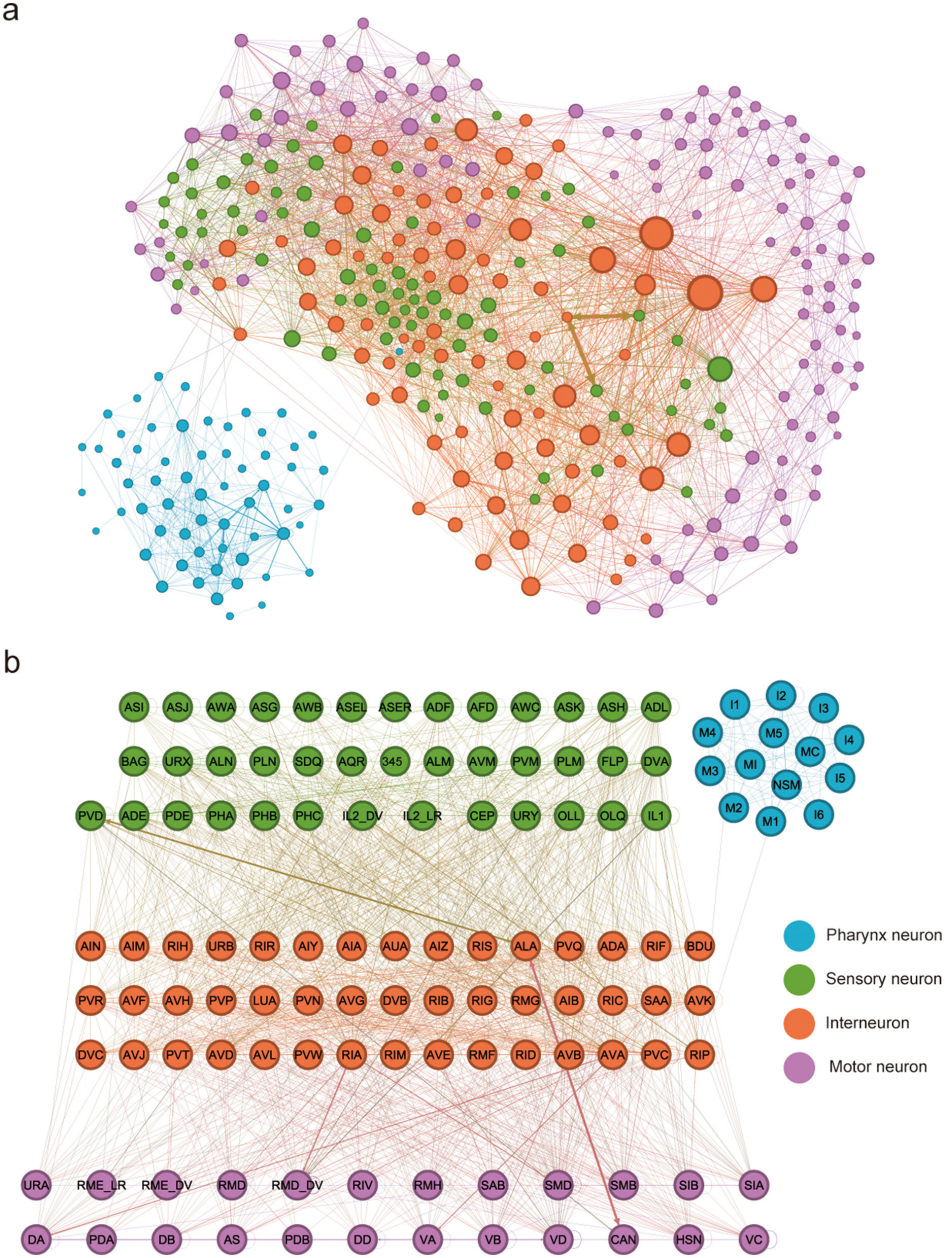
**(a)** The neural network of *Caenorhabditis elegans*. Neuron size reflects its connectivity degree, the number of neurons it connects to. Edge width represents synaptic strength, calculated as the combined count of chemical synapses and gap junctions. To account for directional connectivity, each gap junction was weighted as equivalent to two reciprocal chemical synapses. **(b)** The compressed neural network of *Caenorhabditis elegans*. Data for the figure is provided in Supplementary Table 1.

The *Caenorhabditis elegans* nervous system is one of the most thoroughly characterized biological neural networks, providing a complete map of all neurons and synaptic connections. The adult hermaphrodite has 302 neurons forming approximately 7,000 synapses, including both chemical synapses and gap junctions that mediate electrical coupling (Figure 1a) (Cook Steven et al., 2019).

Although the nervous system of *Caenorhabditis elegans* already has a minimal structure compared to most known biological neural networks, it can be further compressed via systematic abstraction and consolidation. This approach is based on the observation that the neural network contains functionally redundant neuron clusters, characterized by highly similar input-output transformations and neuromodulator expression profiles. By leveraging this redundancy, a functional equivalence framework can be applied: each homogeneous neuronal cluster is collapsed into a single functional node. All synaptic edges originally connected to neurons within a cluster are then merged into the corresponding node under a weight-preserving constraint. Ultimately, structural consolidation streamlines the neural network to only 122 functional nodes and approximately 2,400 synaptic edges, thereby markedly reducing complexity (Figure 1b) (Wang et al., 2025b,c), and in turn generates a compressed neural network that preserves the key topological and dynamical properties of the original network.

Functionally, the nervous system of *Caenorhabditis elegans* is organized into partially segregated layers that align with a sensory-interneuron-motor hierarchy. Specifically, sensory neurons detect environmental cues and internal states; interneurons integrate and transform this sensory information via recurrent loops; and motor neurons mediate coordinated behavioral output. Pharynx neurons are a group of relatively independent neurons, which have a weak connection strength with the main part of the neural network (Ghosh et al., 2016; Izquierdo and Beer, 2013; Cook et al., 2020). Despite the system's apparent simplicity, this structure enables flexible computation through distributed processing rather than centralized control.

**Figure 2:**
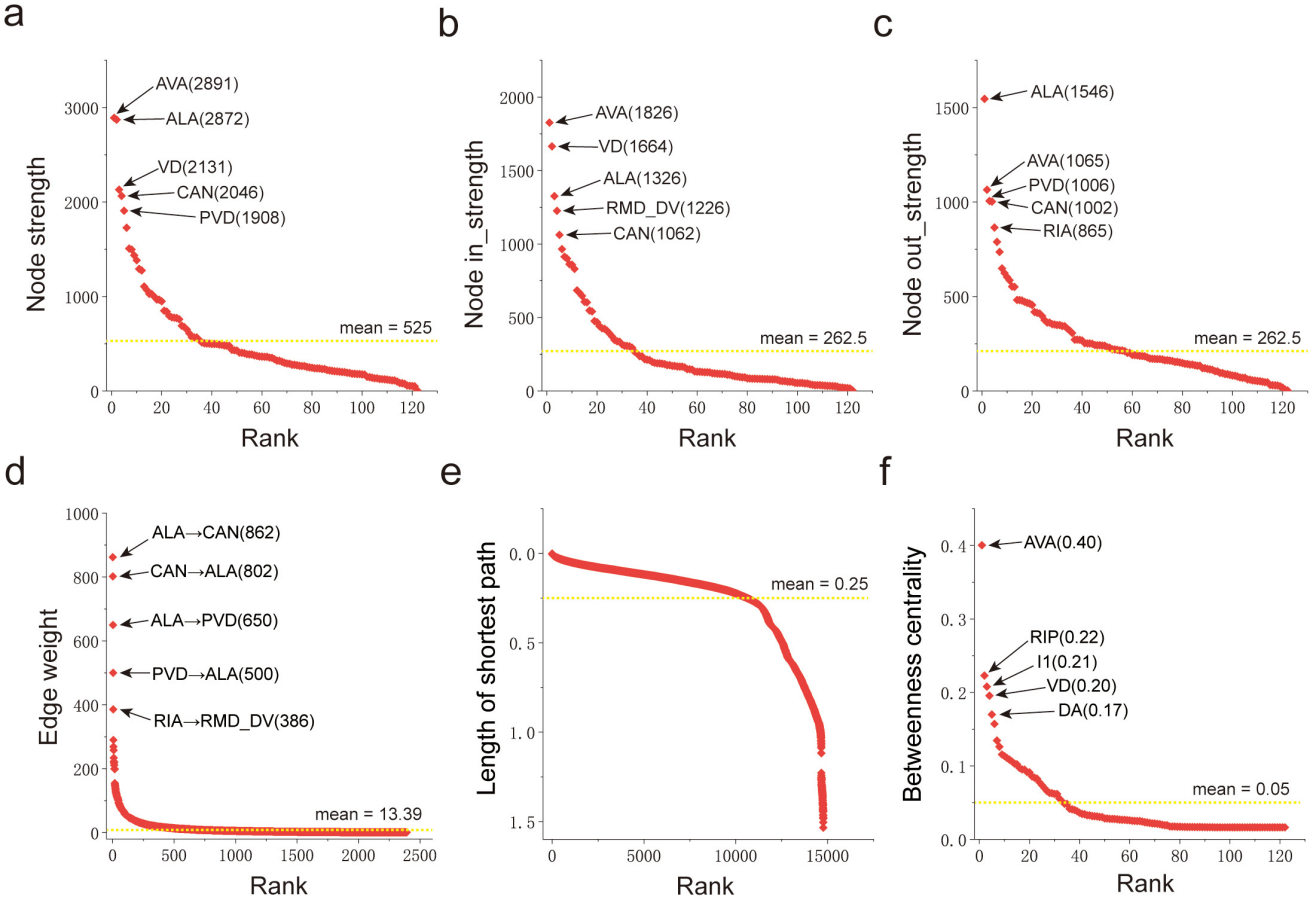
The metrics of *Caenorhabditis elegans* nervous system. **(a)** Node strength of neurons; **(b)** Node in-strength of neurons; **(c)** Node out-strength of neurons; **(d)** Edge weight of synaptic connections; **(e)** Shortest path length between neurons; **(f)** Betweenness centrality of neurons. The dotted lines in the figure represent the average values of the statistical samples.

For the directed and weighted network obtained through the reconstruction of the *Caenorhabditis elegans* connectome, one can employ the network analysis framework in network science to conduct a detailed analysis of its topological organization, weight heterogeneity, and hierarchical-modular structure (Newman, 2004; Mao and Zhang, 2013; Kulig et al., 2015; Opsahl et al., 2010). Figure 2 illustrates the core indicators that characterize the structure of directed and weighted networks in network science: node strength is used to measure the total strength of a node's outgoing and incoming connections; in-strength and out-strength quantify a node's ability to be controlled and to drive (other nodes), respectively; edge weight maps to the functional strength of a connection; shortest path length evaluates the minimum cost of information transmission based on distance cost; and betweenness centrality characterizes a node's hub control potential in information flow across the entire network, based on the frequency of shortest path passage through the node. Statistics are based on the compressed neural network (Figure 1b) of *Caenorhabditis elegans*, and the statistical results are included in Supplementary Table 2.

Analysis of connection strength, in-strength, and out-strength through Figures 2a–c reveals that only a small number of neurons in the *Caenorhabditis elegans* neural network possess high connection strength, suggesting they may be core neurons regulating life activities. This characteristic is consistent with the concept of scale-free networks in network science. Results from the analysis of edge weight (Figure 2d) show that only a small number of connections in this neural network have large weights, further reflecting the heterogeneity of synaptic connections. The shortest path length analysis in Figure 2e indicates that the shortest connection distance between most neurons in the *Caenorhabditis elegans* neural network is short, demonstrating high overall information transmission efficiency of the network. Analysis of betweenness centrality in Figure 2f shows that only a small number of neurons in this neural network exhibit high betweenness centrality. This feature is consistent with the analysis results of neuron connection strength, further confirming that these neurons play a core role in the network and support the stable operation of life activities.

The characteristics analysis of the *Caenorhabditis elegans* neural network shown in Figure 2 indicate that this neural network is small in scale but complete in function, and also possesses the typical structural features of three types of networks: small-world networks (Watts and Strogatz, 1998), scale-free networks (Barabási, 2009), and rich-club networks (Van Den Heuvel and Sporns, 2011). Its core architecture exhibits the coexistence characteristic of dense local connections and a small number of long-range connections. This unique configuration can simultaneously improve information transmission efficiency and enhance network robustness, providing a structural basis for the nervous system to stably perform physiological functions.

The nervous system of *Caenorhabditis elegans* possesses sophisticated dynamic regulatory capabilities, with its core mechanism rooted in neural plasticity and neuromodulatory functions co-mediated by neuropeptides and monoamines. As key signaling molecules, neuropeptides and monoamines serve two primary roles: on the one hand, they provide a modifiable basis for neural circuits, namely neural plasticity, allowing circuits to adjust their properties over time and with experience (Buonomano and Merzenich, 1998; Citri and Malenka, 2008); on the other hand, through neuromodulatory functions, they act directly on the operational process of neural circuits to precisely regulate neuronal activity and signal transmission efficiency. Specifically, this regulation manifests as the ability of neural circuits to flexibly adjust based on two types of key information: first, accumulated experience, such as behavioral memories of chemotaxis and thermotaxis formed when the worm explores its environment; second, internal states, such as physiological conditions like hunger and osmotic stress. The adjustment focuses on three core dimensions: altering the transmission strength of neural signals, optimizing the speed and response mode of signal processing, and adjusting the efficiency of synaptic functional connections between different neurons (Marder and Goaillard, 2006; Piggott et al., 2011; Fang-Yen et al., 2015). Ultimately, this dynamic adjustment enables *Caenorhabditis elegans* to adapt to environmental changes more precisely while maintaining the stability of its own physiological activities.

In summary, the signature computational pattern of the *Caenorhabditis elegans* nervous system is reflected in four key designs: its sparse and modular connection network maintains functional specificity while effectively reducing neuron connection costs; the synergistic effect of mixed chemical and electrical signals not only enables signal transmission but also ensures rapid circuit response and integrates analog and digital information flow modes; the redundant design of synaptic connections between important neurons guarantees the system's anti-interference capability to cope with environmental changes; and the neuropeptide-monoamine mediated plasticity and neuromodulatory mechanisms allow neural circuits to dynamically adjust gain, dynamic characteristics, and coupling strength based on experience or internal states. Together, these principles reveal how a sophisticated biological network achieves complex and adaptable behavioral patterns through efficient and flexible computing capabilities.

## 3 Computational and behavioral mechanisms

The nervous system of *Caenorhabditis elegans* can be regarded as a complex network composed of a series of nested neural circuits (Li et al., 2011; Cho and Sternberg, 2014). To analyze its information processing logic more clearly, a feasible analytical approach is as follows: first, identify specific neural circuits that undertake a complete physiological function from the overall network; then, conduct an analysis of the various properties of these circuits; finally, infer the functional operating principles of the complete nervous system of *Caenorhabditis elegans* based on the analysis results of this subnetwork.

Traditionally, neuroablation has been the primary method for identifying neural circuits associated with specific functions in the intact nervous system (Huang et al., 2025; Ahmed et al., 2011). Its core operation centers on randomly disrupting subsets of neurons through physical ablation, electrical injury, or chemical intervention, followed by assessing the integrity of the target function via behavioral assays, electrophysiological recordings, or other approaches. Through numerous iterative experiments and statistical analyses, the core neuronal populations mediating the function—along with their synaptic connection networks, i.e., the specific neural circuits underlying the function—can be delineated. However, this method is associated with notable limitations. First, it demands extensive control experiments to rule out interference from non-specific damage, leading to substantial labor and material costs. Second, ablation techniques struggle to achieve precise targeting of specific neuronal subtypes, rendering them susceptible to off-target effects.

An feasible strategy leverages post-activation changes in neuronal molecular profiles (Xu et al., 2024; Pérez-Cadahía et al., 2011; Abruzzi et al., 2015). Specifically, high-throughput sequencing is employed to identify transcriptomic differences in individual neurons before and after functional task performance, with neurons exhibiting significant expression alterations defined as the core mediating units of the function. The core principle is that neuronal activation elicits gene expression, with these genes acting as molecular markers for function-associated neurons. Compared to traditional neuroablation, this strategy only necessitates single-cell transcriptomic sequencing of a limited number of samples to screen for core neurons. It not only offers a simpler, more efficient experimental workflow but also facilitates precise correlation between neuronal molecular profiles and functional phenotypes. The neural circuits identification workflow outlined subsequently in this study is built upon this strategy.

As a core mechanism for organisms to adapt to the environment and optimize survival strategies, learning holds crucial biological significance for their survival and evolution. Although *Caenorhabditis elegans* has a structurally streamlined nervous system, it has been shown to possess learning ability to avoid the noxious odor produced by Pseudomonas aeruginosa (PA14) (Martinez and Kesner, 2014; Ha et al., 2010). This aversion behavior provides a clear model for deciphering its neural regulatory mechanisms: Firstly, the chemosensory neurons that mediate the recognition of PA14 odor transmit the sensory signals to downstream interneurons; Subsequently, the interneurons integrate and process the neural signals from all upstream chemosensory neurons, generate motor control signals, and project them to downstream motor neurons; Finally, the motor neurons execute these signals to drive the completion of the aversion behavior.

By combining behavioral experiments with high-throughput RNA sequencing technology, the key genes in the nervous system that regulate the learning process of PA14 noxious odor can be identified; further, relying on the gene expression profiles and structural connection characteristics of individual neurons, the specific neural circuits responsible for this learning behavior in *Caenorhabditis elegans* can be revealed. This neural circuits specifically includes two core components: one is all neurons that perform this learning function to resist the damage caused by the aversive odor of PA14, and the other is the functional synaptic connections formed between these neurons through long-term evolutionary selection.

**Figure 3:**
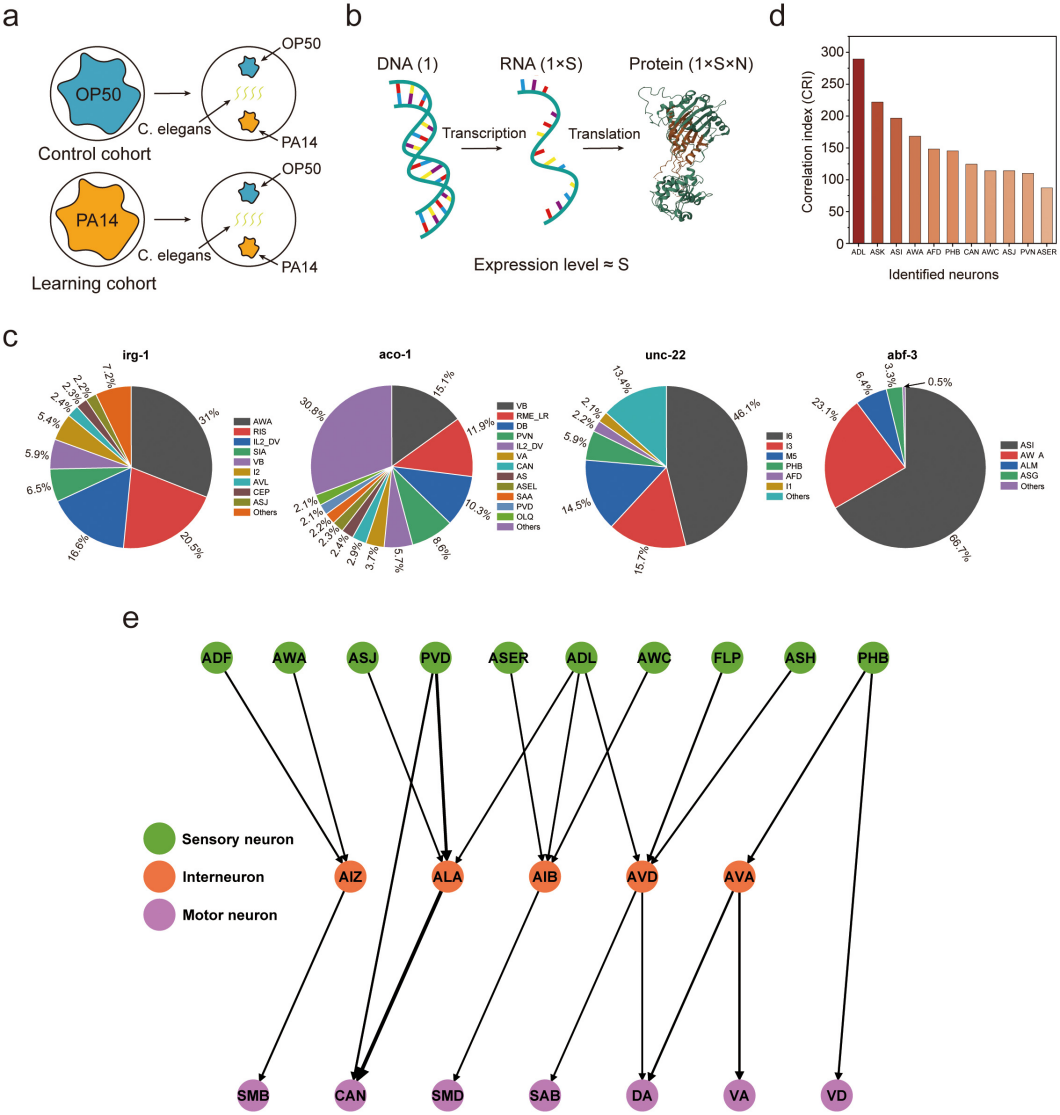
**(a)** Operating principles of the *Caenorhabditis elegans* behavioral experiment for PA14 aversive olfactory avoidance learning. **(b)** Fundamental principles of high-throughput RNA sequencing technology. **(c)** Gene expression proportion in specific functional neurons, excluding those with an expression proportion of less than 2%. **(d)** Identification of functionally associated neurons of *Caenorhabditis elegans* related to PA14 aversive olfactory learning. **(e)** The functional neural circuits related to PA14 aversive olfactory learning in *Caenorhabditis elegans*, which consists of 22 identified functional neurons (10 sensory neurons, 5 interneurons, and 7 motor Neurons) and 21 strong synaptic connections.

The following experimental procedures are intended to outline the design of a behavioral experiment for assessing the avoidance learning ability of *Caenorhabditis elegans* in response to the odor stimulus of pathogenic PA14: At the initial stage of the experiment, two groups of *Caenorhabditis elegans* populations with consistent genetic background and physiological state were both cultured under standard laboratory rearing conditions; subsequently, each group of worms was exposed to two bacterial strains (non-pathogenic Escherichia coli OP50 and pathogenic PA14 strain) respectively, with an exposure duration of 4 hours. After the exposure treatment, each group of worms was transferred to the central area of a new culture plate, where OP50 and PA14 strains had been uniformly spread at two centrosymmetric positions of the plate, thereby establishing a two-choice olfactory stimulation system; finally, the experiment analyzed the worms' preference for the two strains by counting the distribution ratio of each worm group in the OP50 and PA14 strain areas (Figure 3a). The experimental results showed that after the 4-hour learning exposure period (Liu et al., 2022), *Caenorhabditis elegans* could form an aversive memory associated with the odor of the PA14 strain, and the formation of this memory could be verified by their preferential avoidance behavior toward the pathogenic PA14 strain.

High-throughput sequencing technology enables systematic dissection of the molecular mechanisms underlying gene expression regulation during aversive olfactory learning in *Caenorhabditis elegans*. The core principle of this technology is as follows: A single gene in the genome generates a specific number (denoted as S) of RNA molecules through the transcription process; these RNAs further produce proteins via the translation process, and theoretically, the protein yield can be characterized as S × N (where N is a fixed constant reflecting translation efficiency); therefore, at the transcriptional level, the expression level of a gene can be quantitatively characterized by the value of S (Figure 3b). Through differential analysis of gene expression data between the control and learning groups, a total of 338 differentially expressed genes were identified. The high-throughput sequencing data are available in Supplementary Table 3. However, high-throughput sequencing results can only reflect gene expression differences at the whole-worm or tissue-level of *Caenorhabditis elegans*, and cannot accurately localize which specific neuron populations are the functionally responsible units for these gene expression changes.

Previous studies have clearly defined the expression patterns of the aforementioned 338 differentially expressed genes in individual neurons of naive *Caenorhabditis elegans* (Taylor et al., 2021), and Figure 3c presents visualized expression diagrams of some typical genes among them. Based on this, it can be reasonably inferred that during the aversive olfactory learning process for PA14, the expression levels of these 338 differentially expressed genes change in all neurons that express these genes, and the magnitude of this change exhibits a positive correlation with the basal expression levels of these genes in naive *Caenorhabditis elegans*. To achieve quantitative analysis of the activity intensity of functional neurons related to PA14 aversive olfactory learning, this study introduces a core quantitative index, the correlation index (CRI). The formula for calculating the CRI is as follows:


$$CRI_{i}=\sum_{j=1}^{N}\left(W_{ji}\times \left|M_{j}\right|\right).$$ (1)

Herein, *CRI*_*i*_ denotes the correlation index of neuron *i*. *W*_*ji*_ represents the expression proportion of gene *j* in neuron *i* of naive *Caenorhabditis elegans*. *M*_*j*_ stands for the fold change in the expression of gene *j*. *N* refers to the total number of genes that exhibited differential expression following a 4-hour learning exposure period.

After conducting statistical testing and screening analysis on the correlation indices of all neurons, a total of 11 neurons significantly correlated with PA14 aversive olfactory learning were identified (Figure 3d). Notably, most of these 11 neurons are classified as sensory neurons, while the remaining two are one interneuron and one motor neuron. Using the 11 identified PA14 aversive odor learning-associated neurons as the core, integrating the three strongest synaptic connections in the *Caenorhabditis elegans* nervous system, and following the classical processing principle of neural information transmission (“sensory neurons → interneurons → motor neurons”), the neural circuits mediating PA14 aversive odor learning were delineated from the *Caenorhabditis elegans* connectome (Figure 3e) (Wang et al., 2025a). The neural circuits are characterized by simplicity. Specifically, they feature a highly sparse network structure, with a connection density of merely 4% relative to that of fully connected networks.

As shown in the Figure 3e, the functional implementation of PA14 aversive odor learning in *Caenorhabditis elegans* involves the synergistic participation of multiple sensory neurons, interneurons, and motor neurons. Its neural information transmission mode exhibits both the characteristic of ordered flow between hierarchical levels and cross-domain flow across hierarchical levels. The distributed information processing characteristics and flexible information transmission mode presented by the *Caenorhabditis elegans* nervous system are significantly different from those of traditional ANNs. This difference not only provides key clues for in-depth analysis of the working mechanism of the *Caenorhabditis elegans* nervous system but also offers important biological inspiration for the design optimization of ANNs.

## 4 Translation neural circuits to AI design

In the nervous system of *Caenorhabditis elegans*, neural circuits capable of supporting complete functional processes should possess the simplicity and efficiency conferred by protracted evolutionary selection. Existing studies have leveraged the neural characteristics of the *Caenorhabditis elegans* nervous system for the design of ANNs. Compared with traditional ANNs, such bio-inspired ANNs have demonstrated significant performance advantages.

Lechner et al.'s study, taking the nervous system of *Caenorhabditis elegans* as the core biological prototype, proposed an engineering strategy based on its neural circuits (Lechner Mathias et al., 2020). This strategy effectively addressed the key bottlenecks of traditional ANNs in autonomous vehicle control scenarios, namely parameter redundancy, poor interpretability, and insufficient robustness, and provided an important paradigm for the engineering implementation of brain-inspired intelligence.

The core innovation of this study lies in the accurate replication of the hierarchical topology and sparse connectivity characteristics of the *Caenorhabditis elegans* nervous system. The *Caenorhabditis elegans* nervous system achieves complex behaviors such as motor control and environmental navigation through its hierarchical topology. The sparsity of its neural synaptic connections reaches 90%, and there exist strong recurrent synaptic connections between interneurons and command neurons, enabling it to balance information integration efficiency and decision-making response speed simultaneously. Not limited to superficial replication that mimics the number of neurons, the study deeply extracts the functional logic of *Caenorhabditis elegans* neural circuits: through behavioral experiments and connectome analysis, the *Caenorhabditis elegans* nervous system possesses core computational advantages such as distributed control, temporal dynamic adjustment, and maximum information transmission efficiency, which can be migrated directly to the architectural design of autonomous control systems.

In terms of design, the study converted the topological and dynamic characteristics of *Caenorhabditis elegans* neural circuits into a trainable ANN architecture, which specifically includes two core designs:

1. **Neuron model innovation:** a liquid time-constant (LTC) neuron model was adopted. By simulating the dynamic response process of *Caenorhabditis elegans* neurons using continuous-time ordinary differential equations (ODEs), it fundamentally solves the vanishing/exploding gradient problem of traditional recurrent neural networks. Its variable time constant can adaptively match the decision-making response speed in different scenarios, making it more aligned with the real-time characteristics of biological nervous systems.
2. **Network architecture reconstruction:** the front-end uses convolutional layers to extract high-dimensional environmental input features from autonomous driving scenarios, compressing them into 32 feature dimensions, corresponding to the sensory neuron input of *Caenorhabditis elegans*. The control layer constructs a neural circuit strategy module containing only 19 neurons, which accurately replicates *Caenorhabditis elegans* 10 sensory neurons, 5 interneurons, 3 command neurons, and 1 motor neuron. The number of synaptic connections is only 253, with a sparsity 970 fold higher than that of traditional long short-term memory (LSTM) networks. In terms of training logic, the semi-implicit Euler method is used to numerically solve ODEs, enabling end-to-end training while preserving the short-term causal attention mechanism unique to the *Caenorhabditis elegans* nervous system.

In comparative experiments on the autonomous driving lane-keeping task, the performance of this neural circuit strategy further verified its significant advantages over traditional ANNs in terms of parameter efficiency, interpretability, and robustness.

Wang et al.'s study further expanded the engineering application scenarios of *Caenorhabditis elegans* neural principles, extending its application field from the previous autonomous vehicle control to the computer image classification task (Wang et al., 2025a). By accurately deciphering the core working mechanisms of the neural circuits related to *Caenorhabditis elegans* aversive odor learning, this study successfully constructed an ANN for image classification with a compact parameter scale and excellent generalization ability. This provides a new technical pathway for addressing the core pain points of traditional deep learning models, namely parameter explosion, high training costs, and weak scenario adaptability.

Instead of replicating the overall neural system structure of *Caenorhabditis elegans*, the study focused on specific functional neural circuits related to its aversive odor learning. Via behavioral experiments and high-throughput sequencing technology, the study identified a core functional neural circuit of *Caenorhabditis elegans* consisting of 22 neurons and 21 synaptic connections. The synaptic connection sparsity of this neural circuit was significantly higher than that of fully connected networks, demonstrating the compact nature of biological neural circuits. Furthermore, the study translated the information processing mechanism and topological structure of this *Caenorhabditis elegans* core neural circuit into the design of an ANN for image classification, with the core implementation steps as follows:

1. **Topological FUNCTION MATCHING OF NEURAL CIRCUITS:** THe information transmission pathway of sensory neurons (receiving odor stimulus signals) → interneurons (signal integration and processing) → motor neurons (executing avoidance behavior) in the *Caenorhabditis elegans* neural circuit was functionally mapped to the core process of image classification tasks: feature extraction (capturing visual information of images) → feature integration (processing visual features) → category output (outputting classification results). This mapping established a bio-engineering transformation logic.
2. **Reorganization and optimization of network modules:** classic image classification module components, convolutional layers (feature extraction) + pooling layers (feature dimensionality reduction) + fully connected layers (feature integration and output), were used. Module reorganization and connection optimization were performed based on the topological structure and information flow direction of the *Caenorhabditis elegans* core neural circuit, ensuring the network architecture aligned with the efficient processing logic of biological neurons.

In comparative experiments on four public image classification datasets, the *Caenorhabditis elegans* neuro-inspired image classification ANN exhibited significant advantages over traditional image classification models in three core metrics: classification accuracy, consistency of classification accuracy, and model convergence speed. This further verified the key role of the topological structure and information processing logic of biologically derived neural circuits in optimizing the performance of traditional ANNs.

Bardozzo *et al*. proposed the Elegans-AI model, a class of ANNs designed by drawing on the topological structure of the *Caenorhabditis elegans* connectome (Bardozzo et al., 2024). The model aims to enhance the performance and efficiency of ANNs via a bio-inspired network architecture, with three core objectives: (1) translating the natural connectome into an artificial representation; (2) integrating the complex circuit topology of the artificial connectome into deep learning and deep reservoir computing networks to exploit their short-term and long-term memory capabilities; and (3) establishing structural interpretability by analyzing the homophilic/heterophilic properties of the connectome.

Two core model architectures were designed: M1, a Transformer-inspired framework, was employed for image classification tasks on the CIFAR-10 and CIFAR-100 datasets; M2, an autoencoder-inspired architecture, was utilized for the unsupervised image reconstruction task on the MNIST dataset. Both architectures are available in two variants: deep neural network (DNN) and echo state network (ESN). For comparative experiments, three categories of connectomes were constructed: the original *Caenorhabditis elegans* connectome, randomly rewired connectomes, and simulated connectomes.

Experimental results demonstrated that the Elegans-AI model exhibited outstanding performance: M1 achieved a Top-1 accuracy of 99.99% on both the CIFAR-10 and CIFAR-100 datasets, and M2 attained an accuracy of 99.84% in the unsupervised reconstruction task on the MNIST dataset. Additionally, the model demonstrated remarkable parameter efficiency, particularly the ESN variant, for instance, the ESN-based M1 required only approximately 5k trainable parameters on the CIFAR-10 dataset while maintaining a high accuracy comparable to that of the DNN variant. Furthermore, the model based on the original *Caenorhabditis elegans* connectome outperformed those based on randomly rewired and simulated connectomes, validating the critical role of biologically plausible network patterns, small-world properties, and evolutionary optimization in enhancing the learning performance of ANNs.

The research team also extended the deep learning framework to medical image classification tasks on the MEDMNIST2D V2.0 benchmark dataset (Bardozzo et al., 2025; Fiore et al., 2025; Yang et al., 2023). Its core design integrates multiple pre-trained models (including the ResNet series, EfficientNet B0, and VGG19) as multi-scale feature extractors, coupled with a Transformer reservoir structure simulating the pyramidal connectome of *Caenorhabditis elegans*. Across the 12 benchmark tasks of MEDMNIST2D V2.0, the model outperformed existing state-of-the-art (SOTA) models, such as ResNet, Vision Transformer (ViT), and FPViT, on 8 tasks. Particularly notable performance was achieved in tasks including PathMNIST (accuracy: 0.938) and DermaMNIST (accuracy: 0.912). Meanwhile, the model comprises only approximately 50 million trainable parameters and 95,000 non-trainable parameters, which is significantly fewer than that of the ViT series (e.g., ViT-B contains 85 million parameters). This demonstrates a favorable balance between superior performance and parameter efficiency. Notably, the model delivered excellent results without relying on data augmentation techniques, further highlighting its practical applicability.

(Zhao et al. 2024) overcame the limitations of traditional *Caenorhabditis elegans* simulation models and innovatively proposed a full-system simulation framework that integrates its brain neural circuits, somatic structure, and external environment. The core achievements of this work are reflected in two dimensions: model design logic and application value.

The study takes the three dimensions of brain-body-environment as the core of its design, and establishes rigid constraints by extracting key biological features to ensure the biological authenticity of simulation results. In the brain neural dimension, based on the resolved connectome and experimentally validated neurophysiological data, it reproduces neuron morphology, synaptic connection topology, and signal transmission dynamics. In the somatic structure dimension, it focuses on soft-body characteristics, restores muscle fiber distribution and locomotor mechanical properties, and establishes a quantitative mapping of neural excitation-muscle contraction-body displacement. In the external environment dimension, centering on ecological behavior-driven factors, it constructs an interaction model of natural living scenarios to provide authentic external input conditions.

Two aspects underscore the value of this study: On one hand, it drives innovation in neuroscientific research tools for *Caenorhabditis elegans*, which can replace some time-consuming *in vivo* experiments and improve research efficiency. On the other hand, it provides a typical paradigm for bio-inspired AI, with its behavioral control logic of “compact neural circuits + closed-loop interaction”, it can serve as a reference for designing low-parameter, high-robustness ANNs (e.g., robotic navigation and environmental adaptive systems) and offer biological prototypical support for addressing the pain points of traditional AI, namely parameter redundancy and poor interpretability.

The study not only enhances the systematicity and authenticity of *Caenorhabditis elegans* simulation models but also builds an interdisciplinary research bridge between neuroscience and AI. This further provides crucial support for subsequent basic research in related fields and technological applications.

## 5 Discussion

Translating the computational principles of the *Caenorhabditis elegans* nervous system to AI design poses considerable challenges. First, while the *Caenorhabditis elegans* connectome has been fully mapped, the dynamics of synaptic weights, neuromodulatory signaling, and state-dependent plasticity remain poorly characterized. Directly translating anatomical connectivity to AI architectures risks oversimplifying the complexity of biological computation. Second, integrating neuromodulatory mechanisms and context-dependent plasticity into ANNs is non-trivial and often incurs substantial computational overhead. Third, scaling the computational principles from this 302-neuron network to modern AI applications requires rigorous abstraction to preserve computational efficiency while retaining functional relevance.

This study offers a concise review of current academic methodological advances focused on the modification and optimization of ANNs inspired by neural principles in the *Caenorhabditis elegans* nervous system. The objective of biological-inspired design is not to replicate biological systems, but to distill actionable computational principles that enhance learning efficiency, robustness, and adaptability in AI systems. Future work could extend this research framework to Drosophila melanogaster and other organisms with more complex connectomes, thereby further advancing the development of bio-inspired AI.

## 6 Conclusion

The compact, fully characterized nervous system of *Caenorhabditis elegans* provides a unique window into how efficient, adaptive computation emerges from minimal neural circuitry. By investigating its structural motifs, recurrent dynamics, neuromodulatory regulation, and behavioral logic, we can delineate functional principles that inspire novel AI architectures. Translating these principles, rather than replicating anatomical structures, offers a roadmap for developing ANNs that are flexible, robust, and efficient, closing the gap between biological intelligence and machine learning. As research advances in uncovering the dynamic computations of even the smallest biological brains, these insights stand to inform the next generation of brain-inspired AI.
